# A Development of Rapid Whole-Genome Sequencing of *Seoul orthohantavirus* Using a Portable One-Step Amplicon-Based High Accuracy Nanopore System

**DOI:** 10.3390/v15071542

**Published:** 2023-07-13

**Authors:** Kyungmin Park, Juyoung Noh, Kijin Kim, Jongwoo Kim, Hee-Kyung Cho, Seong-Gyu Kim, Eunyoung Yang, Won-Keun Kim, Jin-Won Song

**Affiliations:** 1 Department of Microbiology, College of Medicine, Korea University, Seoul 02841, Republic of Korea; kmpark0131@korea.ac.kr (K.P.); wnduddlk@korea.ac.kr (J.N.); hotdog442@korea.ac.kr (J.K.); chohee98@korea.ac.kr (H.-K.C.); sgagl@korea.ac.kr (S.-G.K.); eyyang@korea.ac.kr (E.Y.); 2 BK21 Graduate Program, Department of Biomedical Sciences, Korea University College of Medicine, Seoul 02841, Republic of Korea; 3 Centre for Infectious Disease Genomics and One Health, Faculty of Health Sciences, Simon Fraser University, Burnaby, BC V5A 1S6, Canada; kijin_kim@sfu.ca; 4 Department of Molecular Biology and Biochemistry, Simon Fraser University, Burnaby, BC V5A 1S6, Canada; 5 Department of Microbiology, College of Medicine, Hallym University, Chuncheon 24252, Republic of Korea; wkkim1061@hallym.ac.kr; 6 Institute of Medical Research, College of Medicine, Hallym University, Chuncheon 24252, Republic of Korea

**Keywords:** Seoul virus, whole-genome sequencing, molecular diagnosis, nanopore sequencing, portable diagnosis

## Abstract

Whole-genome sequencing provides a robust platform for investigating the epidemiology and transmission of emerging viruses. Oxford Nanopore Technologies allows for real-time viral sequencing on a local laptop system for point-of-care testing. *Seoul orthohantavirus* (Seoul virus, SEOV), harbored by *Rattus norvegicus* and *R. rattus*, causes mild hemorrhagic fever with renal syndrome and poses an important threat to public health worldwide. We evaluated the deployable MinION system to obtain high-fidelity entire-length sequences of SEOV for the genome identification of accurate infectious sources and their genetic diversity. One-step amplicon-based nanopore sequencing was performed from SEOV 80–39 specimens with different viral copy numbers and SEOV-positive wild rats. The KU-ONT-SEOV-consensus module was developed to analyze SEOV genomic sequences generated from the nanopore system. Using amplicon-based nanopore sequencing and the KU-ONT-consensus pipeline, we demonstrated novel molecular diagnostics for acquiring full-length SEOV genome sequences, with sufficient read depth in less than 6 h. The consensus sequence accuracy of the SEOV small, medium, and large genomes showed 99.75–100% (for SEOV 80–39 isolate) and 99.62–99.89% (for SEOV-positive rats) identities. This study provides useful insights into on-site diagnostics based on nanopore technology and the genome epidemiology of orthohantaviruses for a quicker response to hantaviral outbreaks.

## 1. Introduction

*Seoul orthohantavirus* (SEOV; family *Hantaviridae*, order *Bunyavirales*) is an enveloped, single-stranded, negative-sense RNA virus that contains small (S), medium (M), and large (L) genome segments [1]. The three RNA genomes encode a nucleocapsid (N) protein in the S segment, two surface glycoproteins (G_n_ and G_c_) in the M segment, and an RNA-dependent RNA polymerase in the L segment [2]. SEOV is a zoonotic pathogen that causes hemorrhagic fever with renal syndrome (HFRS) worldwide, with a mortality rate of <1% [3]. The primary reservoirs of SEOV include brown (*Rattus norvegicus*) and black rats (*R. rattus*), and humans are considered accidental hosts [4]. SEOV infection occurs through the inhalation of aerosolized contaminants or bites from infected rodents [5,6].

Whole-genome sequencing technology provides a robust platform for investigating genome epidemiology across viral populations, which is crucial for understanding evolutionary dynamics and pathogenesis [7,8,9]. The Oxford MinION system (Oxford Nanopore Technologies, ONT), a third-generation sequencer, is a palm-sized portable device that allows real-time viral sequencing for point-of-care testing (POCT) in field situations or hospitals [10,11]. Nanopore sequencing enabled epidemiologists to identify the phylogenetic diversity and geographic distribution of the canine rabies virus collected from countries endemic to outdoor environment [12]. Genome-based diagnosis using nanopore systems has been applied to detect and characterize various emerging viruses, including Ebola virus (EBOV), Zika virus (ZIKV), severe acute respiratory syndrome coronavirus 2, Chikungunya virus, and hepatitis C virus, in clinical specimens [13,14,15]. Using total RNA from virus-infected cells, the one- and two-step reverse-transcription polymerase chain reaction (RT-PCR)-based MinION sequencing approaches were developed for whole-genome sequencing of two New World hantavirus species (Prospect Hill virus and Sin Nombre virus) [16]. Recently, an amplicon-based sequencing method using a portable nanopore system was established to obtain nearly entire-genome sequences of Old World hantavirus species (Hantaan virus) from lung tissues of *Apodemus agrarius* within eight sequencing times [17]. However, to our knowledge, nanopore-based next-generation sequencing (NGS) has not been evaluated to obtain full-length genomic sequences of SEOV.

In the present study, we assessed whether a deployable nanopore-based platform could be used to acquire high-fidelity whole-genome sequences of SEOV for the accurate identification of infectious sources and genetic diversity of the variants. This study provides important insights into the potential application of nanopore sequencing for genome-based diagnostics and the genome epidemiology of orthohantaviruses in the rapid response to hantaviral outbreaks.

## 2. Materials and Methods

### 2.1. Preparation of SEOV RNA

SEOV 80–39 strain was inoculated into Vero E6 cells (ATCC, #DR-L2785). The cells were maintained using Dulbecco’s modified Eagle’s medium (DMEM; Lonza, Basel, Switzerland), including 5% fetal bovine serum (FBS; Gibco, Life Technologies, Carlsbad, CA, USA), 1% HEPES buffer (Lonza), 1% L-glutamine (Lonza), and 0.1% gentamicin (Gibco). The cultures were incubated at 37 °C with 5% CO_2_ and enriched three times at 2-week intervals. Supernatants were harvested for the preparation of SEOV particles. Total RNA was extracted using TRI Reagent LS Solution (Ambion, Austin, TX, USA), according to the manufacturer’s instructions, in the biosafety level-2 (BSL-2) laboratory at Korea University.

Lung tissues of SEOV-positive *R. norvegicus* rats (Rn18-1 and Rn19-5) were aseptically homogenized using a portable microtube homogenizer system (SP Bel-Art, Jersey City, NJ, USA) [18]. Total RNA was extracted from the homogenized tissue specimens using M1 Sample Prep Cartridge Kit (Biomeme, Philadelphia, PA, USA) according to the manufacturer’s instructions.

### 2.2. Quantitative Polymerase Chain Reaction (qPCR)

cDNA was synthesized from 1 µg of total RNA using a High-Capacity RNA-to-cDNA kit (Applied Biosystems, Foster City, CA, USA) with OSM55 (5′-TAG TAG TAG ACT CC-3′). qPCR was conducted using the SYBR Green PCR Master Mix (Applied Biosystems) on a QuantStudio 5 Flex Real-Time PCR System (Applied Biosystems) according to the manufacturer’s instructions. The reaction mixture consisted of 5 μL SYBR Green PCR Master Mix, 0.5 μL of forward and reverse primers (each 5 nM), and 4 μL of diluted cDNA (1:1000 ratio) in a final volume of 10 μL. The SEOV-specific oligonucleotide sequences were SEOV-S719F (forward direction): 5′-TGG CAC TAG CAA AAG ACT GG-3′; and SEOV-S814R (reverse direction): 5′-CAG ATA AAC TCC CAG CAA TAG GA-3’. The cycling conditions were as follows: initial denaturation for 10 min at 95 °C, followed by 45 cycles of 15 s at 95 °C and 1 min at 60 °C. The viral RNA copy number was calculated using the formula for the linear regression curve described previously [19].

### 2.3. Primer Design

All available full-length sequences of SEOV (n = 79 for the S segment, n = 62 for the M segment, and n = 22 for the L segment) from the National Center for Biotechnology Information (NCBI) GenBank (detected until 23 May 2023) were downloaded for the design of universal primers. The viral sequences of the SEOV tripartite genomes were aligned using the ClustalW algorithm in Lasergene (version 5; DNASTAR Inc., Madison, WI, USA). The conserved regions in the alignment were selected as universal primer candidates by the following criteria: amplicon length, approximately 0.8–1.5 kb, and overlaps between amplicons > 70 bp. 

### 2.4. One-Step RT-PCR Amplification

The Superscript™ IV One-step RT-PCR System (Invitrogen, Carlsbad, CA, USA) was used for one-step amplification of SEOV RNA. The viral RNA was enriched using the following mixture: 12.5 μL of 2X Platinum SuperFi RT-PCR Master Mix, 8.75 μL of nuclease-free water, 0.25 μL of Super Script IV RT Mix, 2.5 μL of SEOV-specific universal forward and reverser primers (each 12.5 nM), and 1 μL of total RNA in a final volume of 25 μL. The cycling was conducted on a miniPCR (miniPCR bio, Cambridge, MA, USA) using the following reaction steps: first cDNA synthesis for 30 min at 50 °C and 2 min at 94 °C, followed by 45 cycles of 30 s at 94 °C, 30 s at 45 °C, and 1.5 m at 72 °C, and final elongation for 5 min at 72 °C. The concentration of the PCR products was measured using a NanoDrop spectrophotometer (Invitrogen). Amplicons were pooled into a single mixture for library preparation and nanopore sequencing.

### 2.5. Library Preparation and Nanopore Sequencing

The pooled amplicon library was prepared using a Ligation Sequencing Kit V14 (SQK-LSK114; ONT, London, UK) according to the manufacturer’s instructions. Within 1 h, the libraries were end-prepared, and the adapters were ligated and loaded with a FLO-MIN114 (R10.4) flow cell (ONT). Once 30,000 reads were generated from the raw data, the prepared DNA library was sequenced on a portable MK1B (ONT) device using a local laptop (Apple MacBook Pro, 2021).

### 2.6. Bioinformatic Analysis

The raw signal data were base-called, and adapter sequences were trimmed in real-time using Guppy (v 3.0.3). To enhance data reliability, reads with a Q-score of 8 or higher were included in subsequent analyses. The filtered reads were integrated into a single FASTQ using Porechop (v. 9.0). The data were filtered to discard residual primer and chimera sequences in the following range: 20 bp–2 kb. Consensus sequences were extracted using the KU-ONT-SEOV-consensus module “https://github.com/KijinKims/KU-ONT-SEOV-consensus accessed on 10 July 2023”. This program mapped reads to each segment of the reference genomic sequence of SEOV 80–39. Variants in the genome alignment were called using Medaka “https://github.com/nanoporetech/medaka accessed on 10 July 2023” and filtered based on variant quality and sequencing depth using BCFtools [20]. Genome polishing was performed to discard mechanical indel errors at a homopolymer site when the error reads were minor variants in the alignment. The consensus sequences were generated from called variants based on reference genomic sequences with the following criterion: the position of insufficient coverage depth (under minimum threshold value 50) was excluded and indicated as ‘N’ using BEDtools [21]. 

## 3. Results

### 3.1. Selection of Universal Primers for SEOV

**Figure 1:**
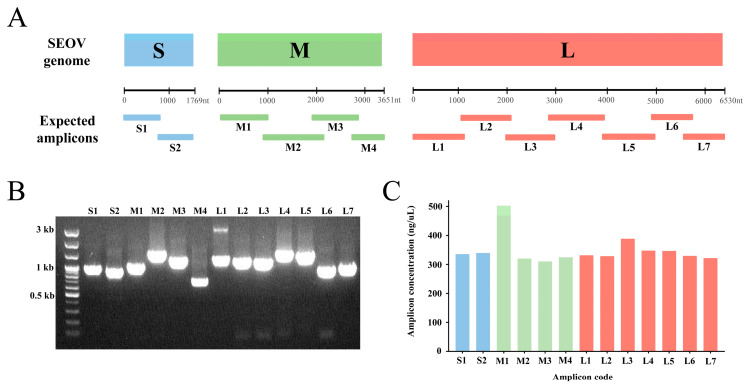
Graphical summary of the universal primer sets and the results of Seoul virus (SEOV) genome amplification. (**A**) The expected size and location of amplicons on the SEOV S, M, and L genomes. (**B**) PCR results of SEOV 80–39 genome amplification. (**C**) The amplification efficiency bias of each primer pair for SEOV.

Based on the alignment of the reference genomes, 13 universal primer pairs that retrieved the complete-length genomic sequences of the SEOV S, M, and L segments were selected and named the SEOV ONT primer set (Figure 1A and Table 1). The specificity of all primer pairs was validated by Sanger sequencing of SEOV 80–39 RNA (Figure 1B). The primers were specific and did not bind to other positions in the SEOV tripartite genome. Amplification bias among amplicons of SEOV genomes was detected (Figure 1C). The M1 amplicon was the most efficient region compared to other polymerase-chain reaction (PCR) products.

### 3.2. Whole-Genome Sequecning of SEOV

**Figure 2:**
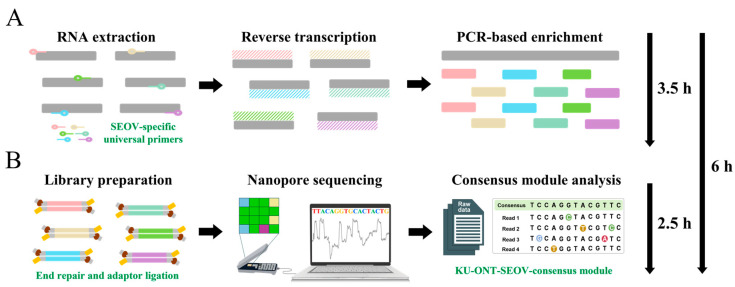
Time-span workflow overview of the one-step RT-PCR-based nanopore sequencing for whole-genome sequencing of Seoul virus (SEOV) within 6 h. The end-to-end workflow included two primary phases: (**A**) the one-step RT-PCR for SEOV genome enrichment from total RNA extraction within the first 3.5 h; (**B**) the high-accuracy nanopore sequencing for SEOV using the KU-ONT-SEOV-consensus module within the next 2.5 h. RT-PCR: reverse-transcription polymerase chain reaction.

A time-span workflow overview of the one-step RT-PCR-based nanopore sequencing for whole-genome sequencing of SEOV is shown in Figure 2. Using amplicon-based nanopore sequencing, nearly full-length SEOV genomic sequences were acquired from SEOV 80–39 RNA samples harboring at least one viral copy (Table 2). With over 1200 × mean depth for each segment, the initial coverage rates of the three SEOV genomes were 98.41% for the S segment, 99.23% for the M segment, and 99.57% for the L segments. The coverage and read depth of SEOV tripartite genomes are shown in Figure S1. To achieve entire-genome sequencing, the termini sequences of the 3′ and 5′ ends were empirically substituted by the conserved region of the family *Hantaviridae*. The accuracy of consensus sequences from MinION sequencing using the KU-ONT-SEOV-consensus module ranged from 99.75 to 100%, as compared to those generated by the Sanger method.

Nearly whole-length SEOV genomic sequences were recovered from lung tissues of SEOV-positive *R. norvegicus* rats (Rn18-1 and Rn19-5) (Table 3). With over 1000 × mean depth for three segments, the initial coverage rates of the tripartite SEOV genomes were 98.42% for the S segment, 99.23% for the M segment, and 99.57% for the L segments. To obtain whole-genome sequencing, the termini sequences of the 3′ and 5′ ends were empirically substituted by the conserved region of the family *Hantaviridae*. The accuracy of consensus sequences from MinION sequencing using the KU-ONT-SEOV-consensus module ranged from 99.62 to 99.89%, as compared to those generated by the Illumina MiSeq platform.

## 4. Discussion

The establishment of a rapid and sensitive diagnostic assay based on genome surveillance is needed in order to investigate genome epidemiology for tracking viral mutations, transmission, spread, and pathogen evolution [22]. Numerous molecular diagnostic methods for SEOV have been evaluated and documented, including loop-mediated isothermal amplification, qPCR, RT-PCR, and NGS [18,23,24,25]. NGS technology is an irreplaceable assay for obtaining precise genome epidemiology that detects and characterizes viral mutations based on massive and high-quality data; however, its application for the molecular diagnosis of pathogens in the field has been limited by sequencer size, slowness, and experimental complexity [26]. As an appropriate platform for POCT of infectious diseases in the field situation, the portable MinION sequencer demonstrated the ability to produce genomic sequences of viruses, including EBOV, ZIKV, Dabie bandavirus, and Hantaan virus, from clinical and animal specimens in real time [15,17,27,28]. In this study, we developed molecular diagnostics to obtain the whole-genome sequences of SEOV using amplicon-based nanopore sequencing within 6 h on a local laptop. To the best of our knowledge, these findings are the first document of portable diagnostic assay for SEOV using a MinION sequencing platform. Our study highlights that the nanopore-based diagnostic approach for SEOV can be used in POCT to monitor and track viral transmission during epidemics or field situations. However, some limitations remain to be further investigated: (1) sensitivity and specificity of the amplicon-based MinION sequencing of SEOV from natural reservoir hosts with highly diverged strains or ultra-low viral copy number; (2) diagnosis performance of the clinical sequencing based on the on-site nanopore system for POCT from patients with SEOV-induced HFRS.

The accuracy of genome sequences generated by high-throughput sequencing plays an important role in the epidemiological surveillance of viral populations based on their genetic and evolutionary diversity [29,30,31]. The nanopore technology offers raw reads with low-quality scores compared to the Illumina system, which generates paired sequence accuracy higher than a Q-score of 30 (99.9%) [32]. Previous studies showed that MinION sequencing with an R9 flow cell (ONT) generates single raw reads with high error rates (approximately 15%) [33,34]. Despite high genome coverage and sequencing depth, nanopore-based approaches have led to the generation of mechanical insertion and deletion (indel) errors that could not be polished naturally in previous R9 chemistry [35,36,37]. The high error rate and indels in the initial nanopore technology were significant limitations to the reliability of subsequent virome analysis. To obtain high-fidelity entire-SEOV-genome sequences, we established a high-quality sequencing protocol with Oxford R10 chemistry and universal primer pairs using the KU-ONT-SEOV-consensus module, which optimizes the nanopore platform to resolve these issues. The accuracy of consensus sequences from the KU-ONT-SEOV-consensus tool ranged from 99.75 to 100% (for SEOV 80–39 isolate) and 99.62 to 99.89% (for SEOV-positive rats) as compared to those generated by the Sanger method and Illumina platform, respectively. These findings demonstrated that amplicon-based nanopore sequencing using the KU-ONT-SEOV-consensus pipeline is suitable for investigating the genetic diversity of SEOV genomes at the variant analysis level.

In conclusion, we developed a portable diagnostic approach to achieve high-fidelity complete-genome sequencing of SEOV to detect infectious sources and their genetic diversity at the variant level using amplicon-based nanopore sequencing. This study provides useful insights into on-site diagnostics based on the nanopore system and genome epidemiology of orthohantaviruses for a quicker response to hantaviral outbreaks.

## Figures and Tables

**Table 1:** Contents of the universal primer set for whole-genome sequencing of the Seoul virus (SEOV) in this study.

Amplicon Code	Primer Name	Sequence (5′ to 3′)	Product Size (bp)
S1	SEOV ONT S1F	TAG TAG TAG ACT CCC TAA ARA G	974
	SEOV ONT S1R	CCA GCA AAC ACC CAT ATT GA	
S2	SEOV ONT S2F	CAA GGT GCA CTT GCA GGR ATG GAG CC	917
	SEOV ONT S2R	TAG TAG TAT GCT CCC TAA AAA GAC	
M1	SEOV ONT M1F	TAG TAG TAG ACT CCG CAA G	1017
	SEOV ONT M1R	GTT GAT GAG TAT GAT GGR ATR CCT GC	
M2	SEOV ONT M2F	TAC CAG ATT TCA GGG CAG ATA GAG GC	1405
	SEOV ONT M2R	CAT ACT CAT AAT CTT TCT CAA AAT GRC	
M3	SEOV ONT M3F	ACA GAR ACT GCA ATT CAG GCA C	1187
	SEOV ONT M3R	TGG AGC CCT ACT TTA CAA GG	
M4	SEOV ONT M4F	CCT TGT AAA GTA GGG CTC CA	1008
	SEOV ONT M4R	TAG TAG TAT GCT CCG C	
L1	SEOV ONT L1F	TAG TAG TAG ACT CCG GAA G	1227
	SEOV ONT L1R	ATT TTC TCK AGG TTC ATG TTG AC	
L2	SEOV ONT L2F	GCC TGC ATT CAA TTT TTA AAC CTC	1150
	SEOV ONT L2R	ATA CCR CTT GCA CCA ACT GTA GA	
L3	SEOV ONT L3F	GTG CAA TCT TTG AYA ACC TAA GGT ATC	1106
	SEOV ONT L3R	GTG AAT CGC CTG AAT TTH GCT G	
L4	SEOV ONT L4F	CAG AAG GCA CAA GCT AGR ATT GT	1352
	SEOV ONT L4R	TCR TGC TGA AAT GTC TCA CCR G	
L5	SEOV ONT L5F	AGT AAG GTT GAG AGA TTA TAT GGG AC	1269
	SEOV ONT L5R	CTT CAA ACC ATA ATG CAT GGA ACC	
L6	SEOV ONT L6F	GTT ATG TAT TGA RGT TTG GAG GTG GGC	868
	SEOV ONT L6R	CAC GAT TCA TTA TTA ATG AAT AAG CAG C	
L7	SEOV ONT L7F	CAT ACT ATC CGT GAT GTT CG	913
	SEOV ONT L7R	TAG TAG TAT GCT CCG GAA RAT G	

**Table 2:** Summary of nanopore sequencing results for Seoul virus (SEOV) from SEOV 80–39 RNA using the KU-ONT-SEOV-consensus module.

SEOV RNA Copy Number (Copies/μL)	Raw Data Size (No. of Total Reads)	Reads Mapped to Reference Sequence (%) ^a^	Segment	Average Depth ^b^	Coverage Rate (%)		Accuracy (%) ^e^
					Initial ^c^	Polished ^d^	
10^4^ to 10^5^	67.2 Mb (29,306)	28,692 (97.9)	S	1396.52	98.42	100	100
			M	2068.61	99.23	100	100
			L	2689.96	99.57	100	99.97
10^3^ to 10^4^	47.3 Mb (29,760)	29,196 (98.1)	S	1357.42	98.42	100	100
			M	1424.74	99.23	100	100
			L	1760.08	99.57	100	99.97
10^2^ to 10^3^	52.7 Mb (28,958)	28,376 (98.0)	S	1229.83	98.42	100	100
			M	1513.11	99.23	100	100
			L	2523.28	99.57	100	99.97
10 to 10^2^	66.5 Mb (29,234)	28,517 (97.5)	S	1507.81	98.42	100	100
			M	1935.45	99.23	100	100
			L	2888.58	99.57	100	99.95
1 to 10	62.8 Mb (29,626)	29,231 (98.7)	S	1461.45	98.42	100	100
			M	1671.79	99.23	100	100
			L	2851.83	99.57	100	99.95
0 to 1	84.6 Mb (29,527)	28,802 (97.5)	S	2181.32	98.42	100	100
			M	2415.84	99.23	100	99.75
			L	1970.87	99.57	100	99.92

^a^ Viral reads mapped to a reference sequence were calculated using the SEOV 80–39 strain generated by Sanger sequencing. ^b^ Sequencing depth was calculated using the formula (average read length × number of reads matching the reference/reference genome size). ^c^ Initial coverage rate was calculated from the raw data of consensus sequences using the KU-ONT-SEOV-consensus module. ^d^ The modified coverage rate was calculated from consensus sequences that were polished using both 3′ and 5′ termini sequence determination. ^e^ The accuracy of SEOV genomic sequences from nanopore sequencing was compared to the SEOV 80–39 strain generated by Sanger sequencing in this study.

**Table 3:** Summary of nanopore sequencing results for the Seoul virus (SEOV) from lung tissues of SEOV-positive rats using the KU-ONT-SEOV-consensus module.

Sample (Tissue)	SEOV RNA Copy Number (Copies/μL)	Raw Data Size (No. of Total Reads)	Reads Mapped to Reference Sequence (%) ^a^	Segment	Average Depth ^b^	Coverage Rate (%)		Accuracy (%) ^e^
						Initial ^c^	Polished ^d^	
Rn18-1 (Lung)	10^5^	79.1 Mb (29,098)	27,822 (95.6)	S	1011.79	98.42	100	99.72
				M	2211.09	99.23	100	99.62
				L	3241.27	99.57	100	99.88
Rn19-5 (Lung)	10^5^	47.3 Mb (29,760)	29,196 (98.1)	S	1357.42	98.42	100	99.80
				M	1424.74	99.23	100	99.81
				L	1760.08	99.57	100	99.89

^a^ Viral reads mapped to a reference sequence were calculated using the SEOV 80–39 strain generated by Sanger sequencing. ^b^ Sequencing depth was calculated using the formula (average read length × number of reads matching the reference/reference genome size). ^c^ Initial coverage rate was calculated from the raw data of consensus sequences using the KU-ONT-SEOV-consensus module. ^d^ The modified coverage rate was calculated from consensus sequences that were polished using both 3′ and 5′ termini sequence determination. ^e^ The accuracy of SEOV genomic sequences from nanopore sequencing was compared to those viral genomic sequences generated by Illumina Miseq platform.

## Data Availability

Not applicable.

## Supplementary Materials

The following supporting information can be downloaded at https://www.mdpi.com/article/10.3390/v15071542/s1: Figure S1: Coverage and read depth of Seoul virus (SEOV) tripartite genomes from SEOV 80–39 RNA by amplicon-based nanopore sequencing.
