# Supplementary material for: A Development of Rapid Whole-Genome Sequencing of Seoul orthohantavirus Using a Portable One-Step Amplicon-Based High Accuracy Nanopore System
Source: Viruses. 2023 Jul 13;15(7):1542. doi: 10.3390/v15071542 (PMC10386077; doi:10.3390/v15071542)
Supplement: Supplementary file 1 [file viruses-15-01542-s001.zip › viruses-2490092-supplementary.pdf]

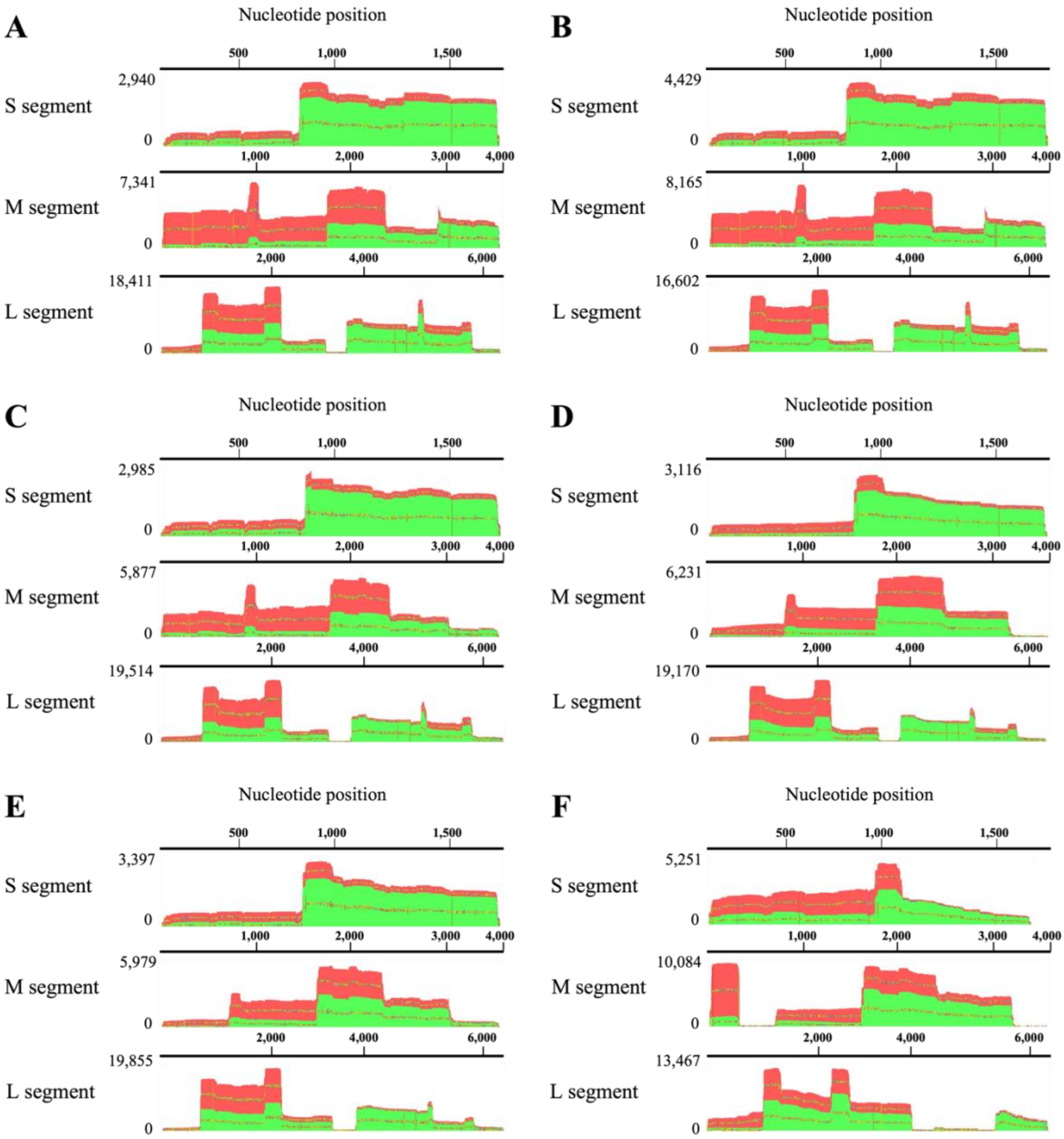

Figure S1. Coverage and read depth of Seoul virus (SEOV) tripartite genomes from SEOV 80-39 RNA by amplicon-based nanopore sequencing. Whole-genome sequencing of SEOV was performed by amplicon-based MinION sequencing within 6 h. The genome coverage and read depth of SEOV are shown. The raw data was analyzed for SEOV 80-39 genomes generated by Sanger sequencing using a reference mapping tool in CLC Genomics Workbench v.22.0.2. (A) 10^4 to 10^5 viral copies/μL of SEOV 80-39 RNA, (B) 10^3 to 10^4 viral copies/μL of SEOV 80-39 RNA, (C) 10^2 to 10^3 viral copies/μL of SEOV 80-39 RNA, (D) 10 to 10^2 viral copies/μL of SEOV 80-39 RNA, (E) 1 to 10 viral copies/μL of SEOV 80-39 RNA, (F) 0 to 1 viral copies/μL of SEOV 80-39 RNA.
